# Effect of Benzothiazoline Ligand and Corresponding Organoantimony(V) Derivative on the Reproductive System of Male Rats

**DOI:** 10.1155/BCA/2006/20979

**Published:** 2006-04-04

**Authors:** D. Shanker, A. K. Rai, Y. P. Singh, H. Rehwani, V. Khushalani, R. S. Gupta

**Affiliations:** ^1^Department of Chemistry, University of Rajasthan, Jaipur 302004, India; ^2^Reproduction Physiology Section, Department of Zoology, University of Rajasthan, Jaipur 302004, India

## Abstract

Benzothiazoline $\text{H}\overset{⎴}{{\text{NC}}_{6}{\text{H}}_{4}\text{SC}}({\text{C}}_{6}{\text{H}}_{5})\text{CH}:\text{C}(\text{OH}){\text{COOCH}}_{3}$ **1** prepared by the condensation reaction of aroyl pyruvate and 2-aminothiophenol has been treated with Ph_3_Sb(OPr^i^)_2_ to yield ${\text{Ph}}_{3}\overset{⎴}{\text{Sb}[{\text{SC}}_{6}{\text{H}}_{4}\text{NC}({\text{C}}_{6}{\text{H}}_{5})\text{CH}:\text{CO}}{\text{COOCH}}_{3}]$ **2**. These compounds have been characterized by elemental analyses and molecular weight determinations. The probable structures of the ligand as well as antimony complex have been tentatively proposed on the basis of IR and NMR (^1^H and ^13^C) spectral evidences. Both compounds have been tested for their antifertility activity in male albino rats. The oral administration of compounds **1** and **2** at the dose level of 10 mg/rat/day significantly reduced the weights of testes, epididymides, ventral prostate, and seminal vesicles. The production of preleptotene spermatocytes was decreased by 36.57%; 57.23%, pachytene spermatocytes by 40.06%; 62.01%, and secondary spermatocytes by 52.45%; 63.22%, following the treatment of compounds **1** and **2**, respectively. The marked reduction in sperm motility and density resulted in infertility by 100%. Significant (*P* < .01) alterations were found in biochemical parameters of reproductive organs in treated animals as compared to control group. It is concluded that all these effects may finally impair the fertility of male rats.

## INTRODUCTION

A large number of antimony(III) compounds have been tested as bactericides [1] and fungicides [2]. The pharmacological activity of antimony compounds has been developed ever since the advent of rational chemotherapy [3, 4]. A large number of antimony compounds have been found to be most effective against various diseases [5, 6]. Early studies sought to develop this element as anticancer compound with the current reports of the *in vitro* cancer properties of diphenylantimony compounds [7, 8]. Phenothiazine and related compounds containing −SC_6_H_4_N− moiety are well known to affect the hypothalamus-pituitary-gonadal axis and thus resulting in a delay in ovulation and menstruation in women [9]. Such type of effects were also observed in rats and dogs [10, 11]. The rate of implantation was lowered and the reduction in litter size has been reported as a result of exposure to some phenothiazine derivatives [12, 13]. Two compounds of benzothiazoline derived from 2-aminothiophenol and *β*-diketone with antimony(III) [14] and aluminium [15] have been tested for antifertility in male rats and were found to show significant antifertility activity.

No comparison of antifertility of benzothiazoline ligand with its metal derivative has been reported so far.

In view of this, we were prompted to synthesize, characterize, and carry out the antifertility activity of ligand derived from aroyl pyruvate and 2-aminothiophenol and their organoantimony(V) derivative. In the present paper, we are reporting the synthesis, characterization, and antifertility activity of these compounds.

## MATERIALS AND METHODS

### Synthesis of compound 1

The benzothiazoline $\text{H}\overset{⎴}{{\text{NC}}_{6}{\text{H}}_{4}\text{SC}}{\text{(C}}_{6}{\text{H}}_{5}{\text{)CH:C(OH)COOCH}}_{3}$ **1** has been synthesized [16] by the equimolar condensation of aroyl pyruvate C_6_H_5_C(O)CH : C(OH)COOCH_3_ [17] with 2-aminothiophenol. This compound has been used for the preparation of organoantimony(V) derivative ${\text{Ph}}_{3}\text{S}\overset{⎴}{\text{b}[{\text{SC}}_{6}{\text{H}}_{4}\text{NC}({\text{C}}_{6}{\text{H}}_{5})\text{CH}:\text{CO}}{\text{COOCH}}_{3}]$.

### Synthesis of compound 2

A weighed amount of sodium metal (0.26 g, 11.31 mM) was added to ∼ 20 ml of well-dried isopropanol and the mixture was stirred for ∼ 1 hour. A benzene solution of Ph_3_SbBr_2_ (2.90 g, 5.65 mM) was added to it. The reaction mixture was refluxed for about one hour. Sodium bromide precipitated during the reaction was filtered off and the removal of excess solvent from the filtrate at reduced pressure yielded a solid Ph_3_Sb(OPr^i^)_2_.

A benzene solution of Ph_3_Sb(OPr^i^)_2_ was added to benzene solution of the ligand ${\text{HNC}}_{6}{\text{H}}_{4}\text{SC}\overset{⎴}{({\text{C}}_{6}{\text{H}}_{5})\text{C}}\text{H}:\text{ C}(\text{OH}){\text{COOCH}}_{3}$ (1.77 g, 5.65 mM). This reaction mixture was refluxed for ∼ 5 hours on a fractionating column. The isopropanol liberated during the course of the reaction was fractionated and estimated periodically [18] to monitor the progress as well as completion of reaction. Then, the excess amount of the solvent was removed under reduced pressure to afford a coloured, viscous compound. For purification, this compound was dissolved in minimum amount of benzene and then pet ether (40–60°C) was added to it till a viscous compound begins to separate. The mixture was placed at −10°C overnight. After decanting off the solvent, a viscous compound was obtained which was finally dried under vacuum. The compound was analyzed [19] to give N = 2.07; S = 4.78%, calc for C_35_H_28_NO_3_SSb; N = 2.11; S = 4.83%. Molecular weight of this compound has been determined (found 642; calc 664) ebullioscopically in benzene solution using Beckman's thermometer.

Proven-fertile male albino rats of the Wistar strain, weighing 150–185 g (90–100 days old), were used. They were housed in steal cages and maintained under standard conditions (12 h light/12 h dark; 25 ± 3°C; 35%–60% relative humidity). Rat feed (Ashirwad Industries Ltd, Chandigarh, India) and water were provided *ad libitum*.

The protocol of the experiments is outlined in Table 1. Body weights of treated rats were taken weekly to ensure their well-being. The rats were cohabitated with proestrous females in 1 : 2 ratio to assess the fertility test by natural mating. The mating exposure tests of compounds **1** and **2** treated animals were performed before and on the 55th day of treatment. Presence of spermatozoa in vaginal smear of the cohabitated females was used as an evidence of mating. On the 16th day laparotomy was performed to note the implantation sites, then females were allowed to complete the term. The number of litters delivered was recorded. Treated animals were anesthetized on the 61st day with solvent ether and their testes, epididymides, ventral prostate, seminal vesicle were dissected out and weighed. Sperm motility in cauda epididymides and sperm density in testes and cauda epididymides were assessed [20]. Blood and serum of experimental rats were analyzed for various parameters (Table 2) [21–28]. The protein, sialic acid, glycogen, fructose, and cholesterol were estimated in testes, epididymides, and accessory sex organs [29–33]. Remaining tissues were fixed in Bouin's fluid. Paraffin sections were made and stained with hematoxylin and eosin. Diameters of seminiferous tubules were measured by using the “camera lucida.” The cell population dynamics was studied for each cell type per crosstubular section. Various testicular cell components were quantitatively analyzed using spherically appearing sections. Abercrombie's correcting factor was introduced [34]. Results were analyzed statistically using Student's “*t*” test.

## RESULTS AND DISCUSSION

Benzothiazoline ligand $\text{H}\overset{⎴}{{\text{NC}}_{6}{\text{H}}_{4}\text{SC}}({\text{C}}_{6}{\text{H}}_{5})\text{CH}:\text{ C}(\text{OH}){\text{COOCH}}_{3}$ has been synthesized by the reaction of aroyl pyruvate with 2-aminothiophenol in 1 : 1 molar ratio:

(1) $${\text{C}}_{6}{\text{H}}_{5}\text{C}(\text{O})\text{CH}:\text{C}(\text{OH}){\text{COOCH}}_{3}+{\text{H}}_{2}{\text{NC}}_{6}{\text{H}}_{4}\text{SH }\overset{{\text{C}}_{6}{\text{H}}_{6}}{\underset{\text{Reflux}}{\to }}\text{ H}\overset{⎴}{{\text{NC}}_{6}{\text{H}}_{4}\text{SC}}({\text{C}}_{6}{\text{H}}_{5})\text{CH }:\text{ C}(\text{OH}){\text{COOCH}}_{3}+{\text{H}}_{2}\text{O}$$.

**Scheme 1:**
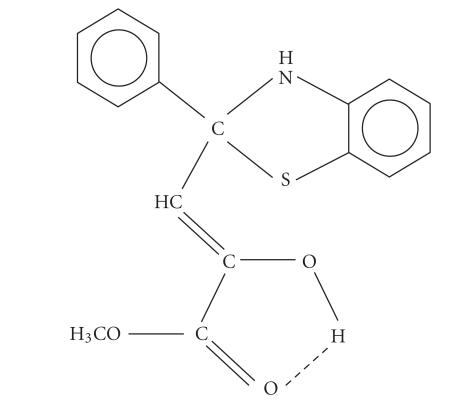
Structure of $\overset{⎴}{{\text{HNC}}_{6}{\text{H}}_{4}\text{S}}{\text{C(C}}_{6}{\text{H}}_{5}{\text{)CH:C(OH)COOCH}}_{3}$.

The water liberated during the course of reaction was removed azeotropically with benzene. This yellow viscous compound was purified by vacuum distillation (108–111°C, 0.1 atm). The spectroscopic [IR, NMR (^1^H and ^13^C)] characterization [16] indicates the presence of benzothiazoline ring and in contrast to the benzothiazolines, $\text{H}\overset{⎴}{{\text{NC}}_{6}{\text{N}}_{4}\text{SC}}({\text{CH}}_{3})\text{C}(\text{O}){\text{CH}}_{3}$ derived from the simple *β*-diketone, enolization of pyruvate residue also takes place due to the hydrogen bonding of enolic OH group with oxygen of ester group, due to which this ligand behaves as bifunctional tridentate ligand during the complexation. The above structure (Scheme 1) has been proposed on the basis of these evidences.

Reaction of this ligand has been carried out with Ph_3_Sb(OPr^i^)_2_ which was prepared by the reaction of NaOPr^i^ and Ph_3_SbBr_2_ in 2 : 1 molar ratio [35] in benzene:

(2) $${\text{Ph}}_{3}\text{Sb}{\left({\text{OPr}}^{\text{i}}\right)}_{2}+\text{H}\overset{⎴}{{\text{NC}}_{6}{\text{H}}_{4}\text{SC}}({\text{C}}_{6}{\text{H}}_{5})\text{CH}:\text{C}(\text{OH}){\text{COOCH}}_{3}\overset{{\text{C}}_{6}{\text{H}}_{6}}{\underset{\text{Reflux}}{\to }}{\text{Ph}}_{3}\text{S}\overset{⎴}{\text{b}[{\text{SC}}_{6}{\text{H}}_{4}\text{N}:\text{C}({\text{C}}_{6}{\text{H}}_{5})\text{CH}:\text{CO}}{\text{COOCH}}_{3}]+2{\mathrm{Pr}}^{\text{i}}\text{OH}↑$$.

This compound, which is viscous dark yellow liquid has been characterized by elemental analysis and probable structure has been proposed on the basis of IR and NMR (^1^H and ^13^C) spectral evidences.

### Spectral studies

#### IR spectrum

A comparative study of the IR spectrum of compound **2** with that of free ligand (compound **1**) shows disappearance of *ν*NH and *ν*OH absorption bands which were observed as broadband at 3252–3420 cm^−1^ and 3421–3630 cm^−1^, respectively, in the spectrum of compound **1**. The presence of *ν*C=N, *ν*Sb−S [36], and *ν*Sb ← N [37] absorption bands at 1606, 425, and 390 cm^−1^ in the spectrum of compound **2** indicates the rearrangement of benzothiazoline ring and subsequent formation of Sb ← N and Sb−S bonds. Deprotonation of OH group during complexation is also supported by the presence of *ν*Sb−O [38] absorption band observed at 755 cm^−1^ in the spectrum of compound **2**. The *ν*Sb−C mode of vibrations appears in the range 449–472 cm^−1^ in the spectrum of compound **2**.

#### ^1^H NMR spectrum

The signals observed at *δ* 4.01 ppm and *δ* 15.1 ppm in the spectrum of free ligand, which have been assigned to NH and OH groups, are found to be absent in the spectrum of compound **2**. Disappearance of these signals indicates the deprotonation of these groups during complexation. The =CH and CH_3_ (ester) group protons have been observed as singlet at *δ* 8.67 ppm and *δ* 2.30 ppm, respectively. The phenylene and phenyl ring protons appear as complex pattern in the range *δ* 7.11–7.94 ppm.

#### ^13^C NMR spectrum

The comparison of the ^13^C NMR spectrum of compound **1** with **2** reveals some useful information about the mode of bonding as well as the geometry of compound **2**. The signal observed at *δ* 158.68 ppm in the spectrum of compound **1** which has been assigned to CN−R group shows downfield shift on complexation. This signal which appears at *δ* 162.97 ppm in the spectrum of compound **2** indicates the rearrangement of benzothiazoline ring during complex formation and subsequent formation of Schiff base derivative with the formation of Sb ← N and Sb−S bonds. Downfield shift in the position of C_1_ and C_2_ carbon signals of −NC_6_H_4_S− group which are appeared at *δ* 153.52 ppm and *δ* 136.7 ppm, respectively, further supports the formation of Sb ← N and Sb−S bonds. The signals of >C=O and =CH groups which appeared at *δ* 166.78 ppm and *δ* 97.78 ppm in the spectrum of **1** also show a downfield shift on complexation indicating the participation of >C−O group in bonding. The signals observed at *δ* 25.76 ppm and *δ* 196.96 ppm have been assigned to CH_3_ (ester) and >C=O (ester) groups, respectively. The −NC_6_H_4_S− group carbon signals appear in the range *δ* 121.79–153.52 ppm. A new set of four signals which appeared in the range *δ* 127.73–152.37 ppm has been assigned to phenyl ring carbons attached to the central antimony atom. The signals for phenyl ring carbons of the ligand moiety of compound **2** have been observed in the range *δ* 126.01–133.73 ppm.

**Scheme 2:**
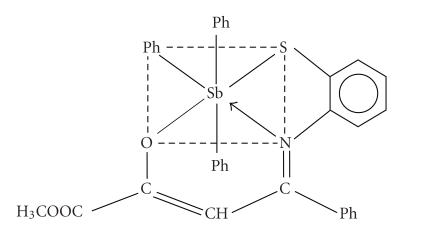
Structure of $\overset{⎴}{{\text{Ph}}_{3}{\text{Sb[SC}}_{6}{\text{H}}_{4}{\text{N:C(C}}_{6}{\text{H}}_{5}\text{)CHC}}{\text{OCOOCH}}_{3}\text{]}$.

On the basis of above evidences, the above structure (Scheme 2) may be tentatively proposed to compound **2**.

The treatment of compounds **1** and **2** did not affect the body weights of treated animals. During the study, all the treated animals showed the normal behaviour and they were healthy in appearance. However, a significant (*P* < .001) reduction was observed in the weights of testes, epididymides, and accessory sex organs (seminal vesicles and ventral prostate) in rats treated with compounds **1** and **2** than those of the control group. This reduction was more significant (*P* < .01) in animals treated with compound **2** as compared to compound **1** treated rats (Table 1). Reduction in reproductive organ weights indicates the low level of androgen. The structural and functional integrities of male reproductive organs are androgen dependent and their weights are used as an index of androgen status of animal [39].

The number of spermatozoa in testes and cauda epididymides was decreased significantly (*P* < .001). Sperm count is considered to be one of the important factors that affect fertility. Low sperm concentration is associated with low fertility. This may be related to decreased testicular size, which may be caused by androgen deprivation [40]. Sperm must be motile to penetrate through the curvival mucus and to migrate through the female genital tract to the site of fertilization. Thus, sperm motility is one of the most important predictors of sperm fertilizing ability. In this investigation, the motility of spermatozoa collected from cauda epididymides was hampered in both compounds **1** and **2** treated groups as compared to control group (Table 3). Sperm motility may be affected by inhibition of adenosine triphosphate (ATP) by uncoupling of oxidative phosphorylation and thus renders the spermatozoa immotile [41]. Suppressed sperm motility and density can be causes of 100% infertility (Table 3).

**Figure 2:**
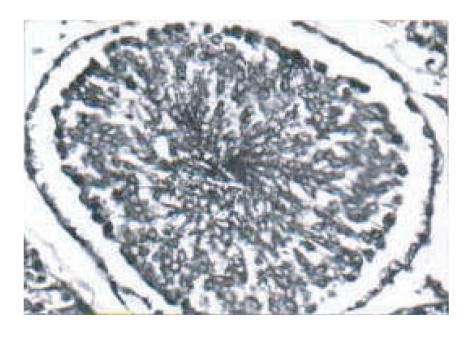
Microphotograph of testis of rat treated with compound **1** showing acute degenerative changes in the histoarchitecture of testis and detachment of germinal epithelial layer. Degeneration of primary spermatocyte stage is seen and lumen is filled with cellular debris. X 200 HE.

**Figure 3:**
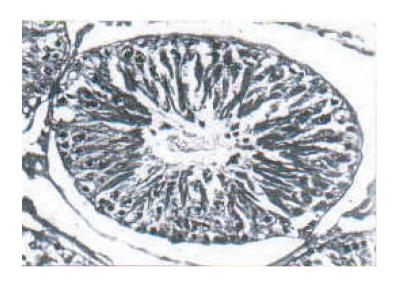
Microphotograph of testis of rat treated with compound **2** showing incomplete spermatogenesis. Seminiferous tubular size is reduced. Secondary spermatocyte stage showing degeneration. Lumen is devoid of sperm. X 200 HE.

A significant decline was noticed in seminiferous tubular diameter following the administration of compounds **1** and **2**. This reflects the tubular shrinkage (Figure 3), which may be due to cell death or sloughing of epithelial cells [42]. The number of spermatogonia was decreased by 24.53%, 49.86%; preleptotene spermatocytes by 36.57%, 57.23%; pachytene spermatocytes by 40.06%, 62.01%, and secondary spermatocytes by 52.45%, 63.22% in compounds **1** and **2** treated animals, respectively (Figures 2 and 3). Sertoli cells perform crucial functions that initiate and maintain spermatogenesis. In these experiments the number of Sertoli cells was decreased significantly (*P* < .001) (Table 4). The decreased number of Sertoli cells may affect the progression of spermatogenesis. This may suggest that spermatogenesis was sluggishly arrested at primary spermatocyte stage.

The treatment of compounds **1** and **2** brought about the alteration in biochemical parameters. The reduction of protein contents in reproductive organs may reflect the alteration in testicular function [43]. The structural integrity of acrosomal membrane is dependent upon sialic acid and due to alteration in its content, the motility and fertilizing capacity of sperm may also be affected [44, 45]. Testicular glycogen was found to be decreased at a significant (*P* < .001) level, it may be correlated to diminished postmeiotic germ cells (secondary spermatocytes and spermatids) which are the site of glucose metabolism [46]. The fructose content of the seminal vesicle was decreased significantly (Table 5) (*P* < .001). It may be suggested that these compounds hamper the glycolitic metabolism of spermatozoa resulting in abnormal sperm function [47]. The significant (*P* < .001) elevation in concentration of testicular cholesterol (Table 5) may indirectly indicate the reduced level of circulating testosterone and thus impairment of spermatogenesis takes place [48]. As far as general metabolism and functioning of vital organs are concerned, all the biochemical parameters (serum and blood) are found within normal range as compared to their control group (Table 2). Our results revealed that both compounds are able to produce antifertility activities in male rats, however, compound **2** is more potent than compound **1**, pertaining to the reproductive organ weight loss, sperm dynamics, and testicular cell population dynamics. The effect of metal derivatives on antifertility activity has been studied [14, 15]. In the present investigation, we studied the effect of antimony(V) derivative, derived from metalation of compound **1**, on antifertility activity which has a more positive effect than compound **1** on male reproductive organs. Similar effects of metal salts on antifertility have been reported earlier [14, 15, 49, 50].

## Figures and Tables

**Figure 1:**
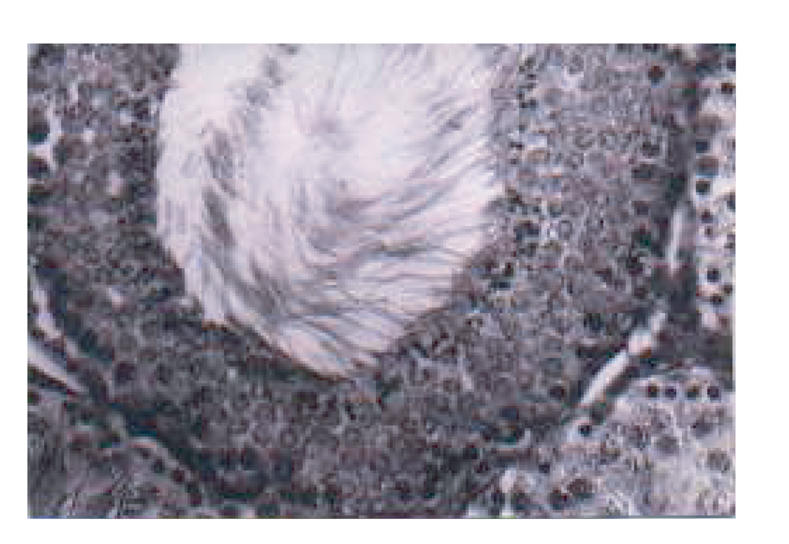
Microphotograph of testis of control rat showing all the successive stages of spermatogenesis. Lumen containing spermatozoa. X 200 HE.

**Table 1:** Effects of compounds **1** and **2** on the body and organ weights (Gr I: rat receiving vehicle (olive oil 0.5 ml/day) gavage orally for 60 days; Gr II: rat treated with compound **1** (10 mg/rat/day) gavage orally for 60 days; Gr III: rat treated with compound **2** (10 mg/rat/day) gavage orally for 60 days; values are mean ± SEM (*n* = 6)).

Treatment	Final body weight (g)	Organ weights (mg/100 g.b.wt)			

		Testes	Epididymides	Seminal vesicles	Ventral prostate

Gr I	250 ± 3.4	1460 ± 22.0	686.45 ± 16.55	720.55 ± 19.0	490.25 ± 26.0
Gr II	222.5 ± 11.5	1211.81 ± 11.11**	560.74 ± 20.77*	704.22 ± 43.41	275.36 ± 0.52**
Gr III	187.5 ± 25.56	1167.86 ± 10.57** ^a^	470.57 ± 10.57** ^a^	565.27 ± 24.44** ^a^	205.91 ± 14.66** ^a^

Level of significance, *P < .01, **P < .001 compared with Gr I (controls).

Level of significance, ^a^ *P* < .01 compared with Gr II (compound **1** treated group).

**Table 2:** Effects of compounds **1** and **2** on blood and serum profile (values are mean ± SEM (*n* = 6)).

	R.B.C.	W.B.C. (mm^3^)	Haemo-	Haematocrit	Blood
Treatment	(million/		globin	value	Sugar	Protein	Cholesterol	Phospholipid	Triglyceride	HDL-Cholesterol
	mm^3^)		(g%)	(%)			(mg/dl)	

Gr I	5.54	8390	14.45	41.50	90.85	13555.54	106.48	118.20	105.60	40.60
Control	±0.10	±55	±0.22	±1.05	±2.45	±222.20	±3.30	±2.67	±5.15	±2.8
Gr II	5.39	8350	13.85	39.00	82.81	13111.09	100.54	111.00	94.17	40.00
D_I_ treated	±0.10	±50	±0.49	±0.86	±4.85	±111.10	±2.28	±3.20	±3.60	±2.20
Gr III	5.42	8275	14.05	38.05	90.62	12666.65	93.05	106.65	92.00	38.70
D_II_ treated	±0.14	±52	±0.12	±1.3	±3.13	±444.40	±2.98	±6.85	±6.00	±2.95

**Table 3:** Sperm dynamics and fertility after the treatment of compounds **1** and **2** (values are mean ± SEM (*n* = 6)).

Treatment	Sperm motility (%) (cauda epididymides)	Sperm density (million/ml)		Implantation sites/ litter delivered		Fertility (%)

		Testes	Cauda	Prefertility	Postfertility	
			epididymides	test	test	

Gr I	65.55 ± 1.98	4.6 ± 0.35	45.15 ± 1.44	10 ± 0.81	10.66 ± 0.47	100
Gr II	19.75 ± 0.66**	2.68 ± 0.20*	10.75 ± 0.90**	10.66 ± 0.47	0	0
Gr III	16.14 ± 1.03** ^a^	1.59 ± 0.28** ^a^	6.65 ± 1.05** ^a^	10.33 ± 0.74	0	0

Level of significance; *P < .01; **P < .001 compared with Gr I (control).

Level of significance; ^a^ *P* < .01 compared with Gr II (compound 1 treated group).

**Table 4:** Testicular cell population dynamics following the treatment of compounds **1** and **2** (values are mean ± SEM (*n* = 6)).

Treatment	Testicular cell counts (number/10 cross-section)					Seminiferous tubular diameter (*μ*m)

	Sertoli cell	Spermatogonia	Preleptotene spermatocytes	Pachytene spermatocytes	Secondary spermatocytes	

Gr I	2.82 ± 0.04	7.58 ± 1.20	20.18 ± 1.85	30.20 ± 1.08	45.60 ± 3.50	276 ± 9.0
Gr II	1.98 ± 0.05**	5.72 ± 0.52	12.8 ± 1.1*	18.10 ± 0.90**	21.68 ± 0.90**	215.2 ± 2.15**
(Percent deviation)^c^	(−29.78%)	(−24.53%)	(−36.57%)	(−40.06%)	(−52.45%)	(−22.02%)
Gr III	1.65 ± 0.10** ^a^	3.80 ± 0.45* ^a^	8.63 ± 0.85** ^a^	11.47 ± 2.2** ^a^	16.77 ± 1.34** ^a^	241.3 ± 2.42*
(Percent deviation)^c^	(−41.48%)	(−49.86%)	(−57.23%)	(−62.01%)	(−63.22%)	(−12.57%)

Level of significance; *P < .01; **P < .001 compared with Gr I (controls).

Level of significance; ^a^ *P* < .01 compared with Gr II (compound **1** treated group).

^c^Values in parentheses are percentage reduction in particular cell type.

**Table 5:** Effects on certain biochemical parameters following the treatment of compounds **1** and **2** (values are mean ± SEM (*n* = 6)).

			Treatment	
		Gr I	Gr II	Gr III

Protein (mg/g)	Testes	210.50 ± 4.15	161.5 ± 2.25**	147.6 ± 3.88** ^a^
	Cauda epididymides	218.00 ± 3.64	180.2 ± 3.32**	161.5 ± 4.40** ^a^
	Seminal vesicles	228.65 ± 2.90	177.75 ± 3.15**	183.05 ± 3.22**
	Ventral prostate	198.05 ± 2.88	164.70 ± 2.9**	157.0 ± 3.10**

Sialic acid (mg/g)	Testes	4.80 ± 0.90	4.30 ± 0.03**	3.92 ± 0.09** ^a^
	Cauda epididymides	5.25 ± 0.11	4.32 ± 0.10**	4.02 ± 0.12**
	Seminal vesicles	4.80 ± 0.13	4.12 ± 0.07*	3.69 ± 0.03** ^b^
	Ventral prostate	5.70 ± 0.10	4.14 ± 0.06**	3.99 ± 0.09**

Glycogen (mg/g)	Testes	3.98 ± 0.11	3.20 ± 0.14*	2.48 ± 0.18** ^a^

Fructose (mg/g)	Testes	4.65 ± 0.10	3.20 ± 0.05**	2.80 ± 0.12** ^b^

Cholesterol (mg/g)	Testes	6.78 ± 0.16	9.18 ± 0.11**	9.56 ± 0.28** ^a^

Level of significance; *P < .01; **P < .001 compared with Gr I (controls).

Level of significance; ^a^ *P* < .01 ^b^ *P* < .001 compared with Gr II (compound **1** treated group).
